# Barriers to and Facilitators of Engagement With Remote Measurement Technology for Managing Health: Systematic Review and Content Analysis of Findings

**DOI:** 10.2196/10480

**Published:** 2018-07-12

**Authors:** Sara Simblett, Ben Greer, Faith Matcham, Hannah Curtis, Ashley Polhemus, José Ferrão, Peter Gamble, Til Wykes

**Affiliations:** ^1^ Institute of Psychiatry, Psychology and Neuroscience Psychology King's College London London United Kingdom; ^2^ MSD IT Global Innovation Center Prague Czech Republic; ^3^ National Institute for Health Research (NIHR) Biomedical Research Centre for Mental Health South London and Maudsley NHS Foundation Trust London United Kingdom

**Keywords:** mHealth, technology, engagement, systematic review, telemedicine, remote sensing technology, patient participation, review

## Abstract

**Background:**

Remote measurement technology refers to the use of mobile health technology to track and measure change in health status in real time as part of a person’s everyday life. With accurate measurement, remote measurement technology offers the opportunity to augment health care by providing personalized, precise, and preemptive interventions that support insight into patterns of health-related behavior and self-management. However, for successful implementation, users need to be engaged in its use.

**Objective:**

Our objective was to systematically review the literature to update and extend the understanding of the key barriers to and facilitators of engagement with and use of remote measurement technology, to guide the development of future remote measurement technology resources.

**Methods:**

We conducted a systematic review using the Preferred Reporting Items for Systematic Reviews and Meta-Analyses guidelines involving original studies dating back to the last systematic review published in 2014. We included studies if they met the following entry criteria: population (people using remote measurement technology approaches to aid management of health), intervention (remote measurement technology system), comparison group (no comparison group specified), outcomes (qualitative or quantitative evaluation of the barriers to and facilitators of engagement with this system), and study design (randomized controlled trials, feasibility studies, and observational studies). We searched 5 databases (MEDLINE, IEEE Xplore, EMBASE, Web of Science, and the Cochrane Library) for articles published from January 2014 to May 2017. Articles were independently screened by 2 researchers. We extracted study characteristics and conducted a content analysis to define emerging themes to synthesize findings. Formal quality assessments were performed to address risk of bias.

**Results:**

A total of 33 studies met inclusion criteria, employing quantitative, qualitative, or mixed-methods designs. Studies were conducted in 10 countries, included male and female participants, with ages ranging from 8 to 95 years, and included both active and passive remote monitoring systems for a diverse range of physical and mental health conditions. However, they were relatively short and had small sample sizes, and reporting of usage statistics was inconsistent. Acceptability of remote measurement technology according to the average percentage of time used (64%-86.5%) and dropout rates (0%-44%) was variable. The barriers and facilitators from the content analysis related to health status, perceived utility and value, motivation, convenience and accessibility, and usability.

**Conclusions:**

The results of this review highlight gaps in the design of studies trialing remote measurement technology, including the use of quantitative assessment of usage and acceptability. Several processes that could facilitate engagement with this technology have been identified and may drive the development of more person-focused remote measurement technology. However, these factors need further testing through carefully designed experimental studies.

**Trial Registration:**

International Prospective Register of Systematic Reviews (PROSPERO) CRD42017060644; https://www.crd.york.ac.uk/PROSPERO/display_record.php?RecordID=60644 (Archived by WebCite at http://www.webcitation.org/70K4mThTr)

## Introduction

Global smartphone ownership has increased, which provides ready access to the internet, and a means of actively logging information and passively gathering big data [1]. Alongside this, a surge in the availability of wearable devices (eg, smart watches and fitness trackers) has enabled continuous and real-time collection of biosignatures and accelerometry [2]. These mobile tools, and platform infrastructures surrounding them, could provide intelligent remote measurement technology (RMT) to support health management. Direct feedback, for instance information about sleep quality, heart rate, mood, and activity, could enable users of RMT to play a more active role in managing their own health that is integrated into daily life. Similarly, feedback to health care professionals could facilitate efficient and timely decisions about treatment. Although these tools have the capacity to augment and extend health care opportunities, they also come with challenges associated with acceptability. A clear understanding of the key barriers to and facilitators of engagement for all stakeholders is an essential part of developing feasible, acceptable, and desired RMT systems.

Engagement is defined as the extent to and manner in which people actively use a resource and has been operationalized as a multistage process involving the point of engagement, a period of sustained engagement, disengagement, and reengagement [3]. Many factors may influence this engagement process at different time points. Indicators of poor engagement may include low initial uptake from the first point of contact or reduced interaction over time, in some cases leading to complete disengagement or dropout. Davis et al [4] conducted a systematic review of the feasibility and acceptability of RMT in primary care from the perspective of staff. They extracted themes from 16 studies, which included concerns regarding changes to roles and responsibilities, the need for extra resources and training, and questions about the usefulness of the data and overtreatment of patients. However, they also highlighted the benefits associated with direct patient education. They emphasized the need for target users, that is, people living with health problems, to be involved in product development and implementation, but the engagement of these target users was beyond the scope of their previous review.

The purpose of this systematic review was to update and extend the understanding of the barriers to and facilitators of engagement with RMT systems for target users. We defined RMT following Davis et al [4], and we categorized it into passive (data are obtained by on-body biosensors and built-in smartphone sensors) and active RMT (requires some interaction, such as completing short questionnaires at repeated time intervals). Passive RMT may interact with active RMT, by sensor activation prompts to perform an action. The review followed the population, intervention, comparison group, outcomes, and study design framework, to answer questions related to barriers to and facilitators of engagement with RMT systems. We achieved this through analysis of the qualitative feedback and quantitative data, such as ratings scales and usage statistics gathered from people using RMT. The aim was to extend the evidence in this area to guide the development of future RMT resources.

## Methods

Following the Preferred Reporting Items for Systematic Reviews and Meta-Analyses (PRISMA) guidelines, we conducted a systematic review of studies to answer the question “What are the barriers to and facilitators of engagement with remote measurement technology?” We registered the trial with the International Prospective Register of Systematic Reviews (PROSPERO registration number CRD42017060644).

### Inclusion Criteria

We included studies if they met the following criteria: (1) were published in English; (2) included health care RMT, defined as any mobile technology that enables monitoring of a person’s health status through a remote interface, with the data then either transmitted to a health care provider for review or to be used as a means of education for the user themselves [4]; and (3) were original studies published from January 2014 reporting the results of questionnaires, interviews, focus groups, and other indicators (eg, reasons for dropout), providing information about barriers to and facilitators of engagement with RMT systems using mHealth tools. We stipulated no diagnostic exclusions, so we included people using RMT to support any physical or mental health condition and healthy populations where interventions focused on improving general well-being.

### Search Strategy

We searched Ovid MEDLINE, IEEE Xplore, EMBASE, Web of Science, and the Cochrane Library using the combined terms “remote” or “mobile” and “technology” or “devices,” along with “telemedicine” and “mHealth.” Multimedia Appendix 1 provides details of all search strategies. The initial search was completed in July 2016 and the process was repeated in May 2017. Two authors (SS and FM) independently screened articles by titles, abstracts, and then full texts to assess whether they met the inclusion criteria. The repeated screening on the second batch of articles was carried out by 2 other authors (BG and HC).

### Data Abstraction and Synthesis

#### Study Characteristics

We extracted the following data: (1) device type and RMT system (including active and passive data); (2) population characteristics, including diagnostic categories, sample size, time using RMT, and the country in which the study was conducted; and (3) methods used to gather qualitative information on the feasibility and acceptability, grouped as follows: usage statistics, questionnaires, structured or semistructured interviews, focus groups, and descriptive feedback.

#### Content Analysis

One author (SS) read and reread the results reported in articles published from January 2014 to July 2016 to extract individual barriers and facilitators (defined as “a circumstance or obstacle that may prevent the adoption of remote measurement technology” or “make adoption easy or easier”). The coding frame was developed by 3 authors (SS, BG, and HC) using these data. It consisted of the following themes: health status, usability, convenience and accessibility, perceived utility, and motivation, with subthemes. This coding frame was then tested on a further batch of articles published from June 2016 to May 2017 (coded by authors BG and HC and discrepancies evaluated by SS). This replication test allowed for a validation and potential extension of the initial coding frame.

Multimedia Appendix 2 and Multimedia Appendix 3 provide an overview of all coded barriers and facilitators. Some subthemes were mentioned as both a barrier and a facilitator depending on circumstances, and were coded separately. Multimedia Appendix 4 summarizes all quotes extracted and coded from each of the articles.

### Assessing Study Quality

Methodological quality was assessed by 2 independent raters using the Mixed Methods Appraisal Tool (MMAT) [5]. The MMAT is a 21-item checklist of 5 research designs, with scores ranging from 0 to 1 in increments of 0.25. The MMAT does not provide a categorical distinction between studies of low or high quality; rather, it provides a descriptive framework of study quality. Interrater reliability has been reported to range from moderate to perfect (kappa range .53-1; Pace et al [5]).

## Results

### Study Selection

**Figure 1:**
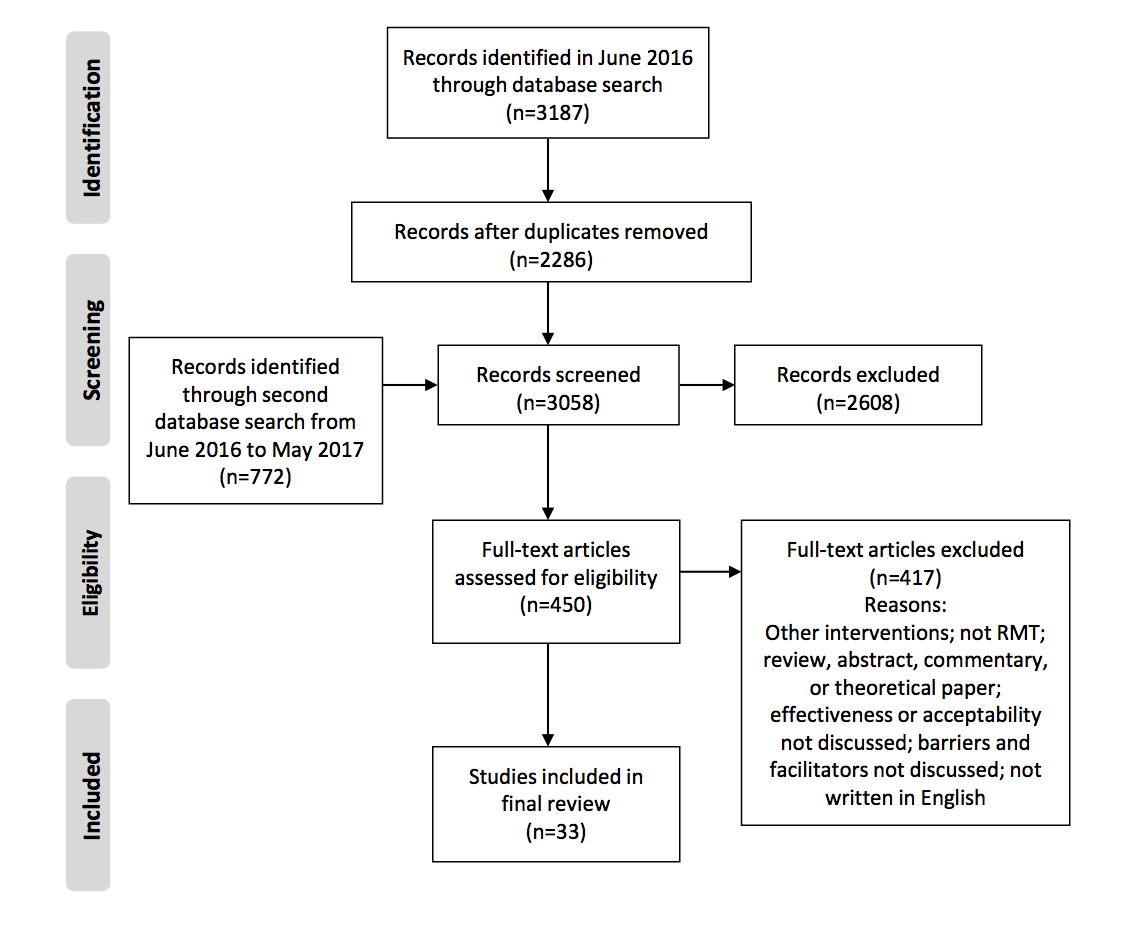
Preferred Reporting Items for Systematic Reviews and Meta-Analyses (PRISMA) flow diagram of study selection. RMT: remote measurement technology.

Of the 3187 abstracts and titles identified, 33 original articles met our inclusion criteria (see the PRISMA flow diagram in Figure 1 for a breakdown of this process). Multimedia Appendix 5 [6-38] presents study characteristics and participant demographics.

### Participants

Studies varied in their sample size (7-365 participants), as well as the age (8-95 years) and sex of participants (30 studies included both male and female participants).

### Study Characteristics

Studies were conducted in 10 countries: the United States (n=24), United Kingdom (n=1), Canada (n=1), Taiwan (n=1), Sweden (n=1), Poland (n=1), Australia (n=1), Switzerland (n=1), Germany (n=1), and New Zealand (n=1). Study durations ranged from 1 to 13 months, and 3 studies consisted of only a single individual or group session.

### Remote Measurement Technology Characteristics

A total of 6 studies used passive RMT, including wearable pedometers and accelerometers, and built-in smartphone activity monitors (see Multimedia Appendix 5). Most studies used active RMT (n=17), including smartphone-based systems (eg, ecological momentary assessment, patient-reported outcome measures, and activity logs) and wireless monitoring devices (eg, blood pressure monitors and weight scales). Both active and passive RMT were used in 10 studies.

RMT systems provided feedback to users (n=17), members of the users’ health care team (n=7), or both (n=9). Feedback was provided in various forms, including visual displays (eg, graphs), report summaries, historic reporting patterns, and messages (eg, health advice and motivational feedback).

### Health Conditions

The studies covered many health conditions, with most concentrating on 1 condition (n=17). A total of 2 studies featured more than 1 physical health diagnosis (diabetes and obesity, and multiple genetic blood disorders). Only 4 studies related to mental health conditions such as psychosis and posttraumatic stress disorder, and 2 studies included both physical and mental health conditions (eg, depression and type 2 diabetes, HIV, and substance use disorders). The remaining studies supported general health and well-being (n=7), and smoking cessation (n=1).

### Assessment of Outcomes

In total, 27 studies employed quantitative methods to identify barriers to and facilitators of using RMT systems, including usage statistics (n=20) and questionnaires (n=19). Most questionnaires (15/19, 79%) were unvalidated measures developed for the study. Only 4 studies used validated measures, including the System Usability Scale, the Telehealth Usability Questionnaire, and the Technology Acceptance Model Questionnaire. Similarly, types of usage statistics reported varied greatly between studies. Of these 27 studies, 9 employed a mixed-methods design and asked for qualitative information (ie, from semistructured interviews and focus groups) and quantitative information from their users; 6 studies employed purely qualitative methods.

### Study Quality

Of the reviewed studies, 2 obtained the maximum score of 1 on the MMAT [6,7], with the remaining studies scoring 0.75 (n=13), 0.5 (n=11), or 0.25 (n=7). Higher ratings were prohibited for a range of reasons, including a lack of adequately reported information regarding researchers’ influence on the qualitative findings and their generalizability, description of sampling method, and method of analysis.

### Quantitative Measures: Engagement and Adherence

Of the 5 studies that reported on the average number of times the RMT system was used, 3 reported the total number of interactions and 2 reported the number of days that people interacted with the app; 2 reported on the percentage of people who wore the wearable device for the whole study; and 4 set a threshold for the appropriate level of adherence (which varied between studies) and reported the percentage of people meeting these requirements. The remaining studies reported idiosyncratic usage statistics that were not comparable across studies. This variability severely limited quantified conclusions. For the few studies that reported the average percentage of time used, this ranged from 64% to 86.5% [8-10]. The average total number of interactions varied between 8.5 and 29.7 and may have depended on the type and length of the intervention [6,11,12]; the lowest level of interaction was with video content and the highest was with a person via a text message. The average numbers of interactions per week also varied between 3.5 [13] and 12 times per week [14]. The average percentage of people who wore the wearable device for the duration of the study ranged from 50% to 75% [15,16], and the percentage of people meeting a prespecified threshold for adherence varied from 41.7% to 81.8% [7,9,12,17]. Although studies reported varying degrees of attrition [10,13,18], dropout rate reporting was more frequent and ranged from 0% to 44% with a mean of 11.0% (SD 11.4). Table 1 summarizes the reasons reported for dropout. Overall, there was significant variation across studies, and there was no specific measure that is comparable across studies.

**Table 1:** Reasons for dropout across studies.

Reason for dropout	Frequency	Related theme
Lost or stolen smartphone	23	Usability
Technical malfunction (eg, smartphone corrupted, not receiving texts, or delivery delays)	7	Usability
Exacerbation of health condition, including participants who were injured or died during the course of the study	6	Health status
Deleted app	3	Usability
App not compatible with existing smartphone	3	Convenience and accessibility
Unexpected usage patterns (eg, switched smartphone off in between answering surveys, left smartphone plugged into charger, used smartphone in airplane mode)	3	Perceived utility
Moved out of area or was discharged from hospital	3	Convenience and accessibility
Sold smartphone	2	Perceived utility
Changed mobile phone or service plan	2	Convenience and accessibility
Practical technical difficulties (eg, not being able to download the app)	2	Usability
Broken smartphone	1	Usability
Inconsistent wireless network	1	Convenience and accessibility
App consumed too much battery	1	Usability
System too slow	1	Usability
Unspecified reason	11	Not applicable

### Qualitative Analysis: Themes of Barriers and Facilitators

We divided themes into 5 major categories that made up a coding frame for structuring the minor themes. The two batches of articles (2014-2015 and 2016-2017) yielded subthemes that fitted within the same coding frame, with all major themes represented across the two time periods providing evidence of validity. No new themes arose in the later studies. The following section describes the findings for each major theme, with barriers and facilitators in italics. Multimedia Appendix 2 and Multimedia Appendix 3 display the categorization of subthemes for active RMT and passive RMT (including combinations of active and passive RMT), respectively.

#### Health Status

*Exacerbations in health conditions*, such as a chronic heart or respiratory condition, or episodes of being acutely unwell, such as experiencing a sickle cell crisis, have been reported to disrupt engagement and RMT use [6,10,18]. This disruption was related to a change in environment (hospital rather than own home) [6,18], as well as the acute exacerbation of health problems. Other longer-term health-related barriers to engagement in RMT included difficulties due to *poor vision* [19]. This was discussed in the context of older age; however, this was not tested directly.

#### Usability

*Technical malfunctions* were by far the most widely reported barriers, with 11 studies reporting ways in which these factors affected usability of the RMT systems [6,10,17,20-27]. This included not receiving notifications or receiving them at the wrong time, disappearance of the app, freezing of the system, losing power or restarting without warning, and difficulties connecting remote (wearable and other smart technology) devices with apps. Studies reported that this led to participant withdrawal [6], data loss [17,23,24], or significantly fewer data entries (eg, by 35%) [10].

Ben-Zeev et al [8] reported that *clarity of information* enhanced usability and facilitated engagement. In their study, 90% of participants reported that they thought they could learn to use the app very quickly, but no data were provided to suggest that these self-reports were valid. For other studies, difficulties inputting information into apps was a reason for discontinuing [15]. This may have depended on the type of data, length of time that participants were required to log data, or the value that people placed on the feedback, but a theme around engagement being potentially facilitated by clear and simple tasks emerged.

Where technical malfunctions and complexities in terms of usability arose, practical support was sometimes necessary. Some studies reported that problems such as “creating user accounts, answering intake question and navigating content due to unexpected behavior of keyboards, scroll bars, buttons, and other interface widgets” could be addressed with minor adjustments [22], although the authors provided no data on changes that had improved engagement. Engelhard et al [9] reported that where technical difficulties arose all could be solved by a phone call with the study coordinator; these authors offered no data to back up this claim.

In addition to technical functionality and clarity of information, we grouped other subthemes under the broader theme of usability. *Speed of the system* was a potential influence on engagement, with 1 participant withdrawing from a study due to frustrations with the slowness of the system [6]. *Use of larger devices* (smart tablets vs smartphones) in 1 study resulted in significantly more diary entries (by 30%) [10]. Given that this difference emerged between 2 groups, it is unclear whether this arose from individual preferences or that larger devices led to better engagement. However, in another study that compared within-group differences, only 20% (10/51) of participants aged between 50 and 94 years were reported to be capable of using a smartphone, as opposed to a larger smart tablet for data entry and active monitoring; of these 10 people, only 3 considered the smaller device easy to use [28]. *Lost or damaged device* was a clear barrier to usability and participation, mentioned in 4 studies [8,13,15,26]. Further disruptions to response collection due to *changes in service plans* such that participants could no longer receive text messages [17] or excessive consumption of the *smartphone battery* [29] were mentioned as a barrier to data entry completion in another study [10].

#### Convenience and Accessibility

*Compatibility with one’s existing routine*, including the ability to use your own devices, appeared as a subtheme. Ding et al [29] reported that 2 participants withdrew because the app was unable to function on their personal smartphone. Peng et al [30] stated that, even though the app functioned correctly, participants did not necessarily use it if other strategies, such as paper logbooks, already satisfied their needs. What is not clear from this study is at what point participants disengaged: immediately or after a trial period? Convenience was limited when there were *restrictions on the placement* of the wearable device—for example, participants had to carry their smartphone in their pocket [31]. However, resulting data loss was not reported. Systems that provided opportunities for *passive or automatic* data collection were endorsed as being more convenient where this approach met the objectives of RMT [32], but the impact on adherence was not a focus of the study.

Where users were required to actively engage with data collection (active RMT), the *presence of notifications* facilitated engagement [14,20]. These notifications became less important once the monitoring had become part of the participant’s daily routine [14]. Surveys were much more likely to be completed if users were prompted with a notification. For instance, 93.5% of check-in surveys were completed following a notification rather than being self-initiated [12]. But other systems seemed to be able to produce high engagement even from self-initiated reports without prompts. For instance, a study by Ben-Zeev and colleagues achieved 62.5% adherence to data collected on mood, sleep, medication use, and psychosis symptoms [8]. But notifications can also be a barrier when they are not received at the right time; Cushing et al [20] and Juengst et al [23] and other studies reported that participants requested the ability to postpone responses to notifications so they might answer them at a convenient time [33], but there is no evidence that when this was done there was an improvement in engagement.

Other major barriers were related to participants’ access to resources such as websites and videos due to a poor internet connection or lack of a Wi-Fi connection, and use of old computer systems [10,11,15]. This caused difficulties with specific processes such as setting up resources [21], with 2 participants withdrawing due to difficulties in acquiring a consistent wireless service [14]. Other problems with accessibility included *poor telephone network coverage*, which caused delays in receiving text messages [15] and, in 1 case, resulted in 39% of participants missing training sessions [7].

*Lack of familiarity with and knowledge* about how to use technology, such as websites, smartphone apps, and wearable devices, was reported as a challenge with using RMT systems and a source of frustration for participants [21,26]. But the impact on engagement was not quantified. *Forgetfulness* was raised as interfering with the individual’s ability to access passwords, complete questionnaires, wear their device, and sync their wearable device to their smartphone [10,16,21,26], but this was not quantified. Digital literacy and other practical barriers were overcome through offering instructions and *support* from the study coordinator [9,28]. Research into the type of support necessary to increase engagement was lacking and may be a subject for future reviews.

Other barriers within this theme included RMT systems not being adequately *tailored* to the disability status of individual participants. In the study of Engelhard et al [9], some participants felt that questions were irrelevant to them and did not want to continue reporting symptoms that showed no sign of change. The authors suggested integrating adaptive patient-reported outcome measures. Cultural relevance of study support materials was also reported to enhance engagement [25]; however, this was a qualitative study that provided no evidence of how it enhanced engagement.

#### Perceived Utility

##### Perceived Rewards

The results of 4 studies demonstrated a positive and motivating effect of feedback [11,32,34,35]. Buchem et al [34] reported that 50% of participants felt motivated by virtual rewards such as badges (ie, an indicator of accomplishment, skill, quality, or interest that can be earned). Dale et al [11] reported that 67% of participants liked receiving motivational texts from the RMT system. The results were less clear in the remaining studies, but some participants reported a benefit associated with learning about their real-time activity [32] and talking about app data with a study coordinator [35].

Further *incentives* that were suggested to increase motivation to engage included social sharing and comparison [16,32,36] or gaming features, including monetary rewards [20]. Another aspect reported to be “enjoyable” in 1 study was the receiving the training instructions, which was seen to be an important contributor toward increased engagement [34].

##### Perceived Costs

*Financial costs* were a clear barrier to engagement in 2 studies. Ho et al [36] found that 56% of their sample, based on their current income, would have struggled to afford a program that required payment of a large initial sum, followed by smaller regular payments. Naslund et al [16] reported that commercially available, wearable tracking devices alone were seen to be expensive and difficult to obtain for individuals with a low income. Some participants who were provided with devices that were perceived to be expensive were found to sell or pawn them [13].

*Privacy* concerns were also reported in 1 study, in which a participant disengaged and switched their mobile phone to airplane mode due to concerns about being tracked [13]. This study investigated an RMT system for people with psychosis and was the only study to raise concerns about privacy as the reason for disengagement. Disengagement was, however, raised in relation to other issues such as feeling uncertain about the user benefits and the *reliability or accuracy* of the data being recorded [15,28].

#### Motivation

The value of the RMT system appeared to be affected by people’s *intrinsic motivation* to learn and sustain engagement. The impact of perceived rewards on motivation has already been mentioned, but these studies did not quantify this effect or report the impact across time. One additional study highlighted that, over time, active RMT became burdensome, and this affected 1 participant’s motivation to engage [30]. Others reported that boredom had a negative impact on engagement [32]. The magnitude of this negative impact was not measured and discussed. *Extrinsic motivation* and reception from others (eg, clinicians) also affected use, with participants reporting a reluctance to try mHealth technologies if their doctor did not recommend it [30]. However, this finding was reported in the context of a hypothetical scenario rather than in a trial of an actual RMT.

### Relationship Between Adherence and Themes

Dropout is a clear indicator of problems with engagement. Reasons for dropout spanned several of the qualitative themes, with problems related to usability of the wearable device and the smartphones apps being the most frequent. Convenience and accessibility was the second most frequent theme. The study that reported the greatest percentage of dropouts included one of the largest samples (n=342) and followed people with a diagnosis of psychosis for 6 months. Studies that reported no dropouts or the odd person dropping out were much smaller (ranging from 8 to 51 participants), and dropout may not be possible to understand here, as the sample might have been highly selective. There was no significant relationship between the percentage of people who dropped out and the length of the intervention in days (*r*_29_=.19, *P*=.31).

A total of 10 studies reported on the impact of variables on adherence in terms of compliance and use of an mHealth device over time. The themes included health status, with greater physical disability [9] and mental health problems (symptoms of posttraumatic stress disorder) [17] being associated with better engagement (ie, participants exceeded usage requirements and provided more responses, respectively), but rehospitalization being a barrier to engagement [13,18]. Issues to do with usability was the second most common category, with technical difficulties accounting for poorer compliance (eg, missed assessments) [10,23], and use of larger mobile tablet, as compared with a smartphone, being significantly higher [10,28]. Confidence in one’s ability to maintain an exercise regimen correlated with percentage of ecological momentary assessment responses [33]. Sociodemographic factors have also been found to influence use of mHealth technology, with age appearing to moderate use [10,28]. Lower household income, higher level of education, and male sex have been found to be facilitators for mHealth technology use [9,28,34].

## Discussion

### Factors Driving Engagement

**Figure 2:**
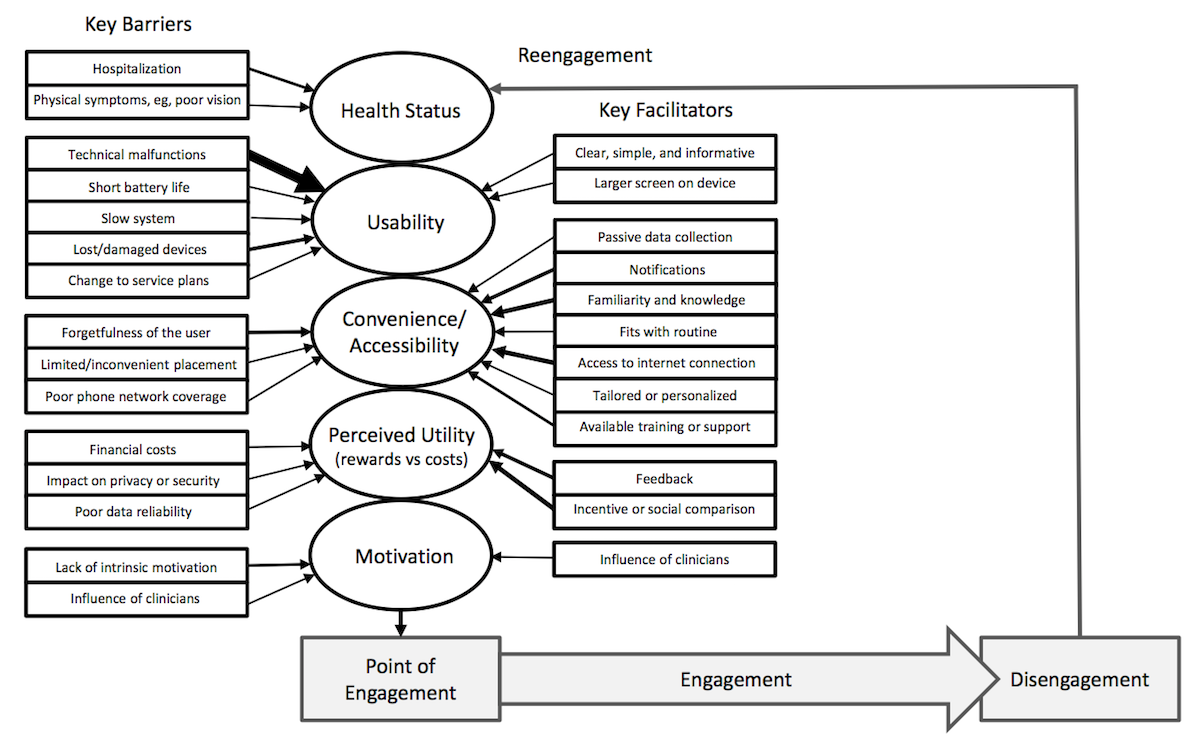
Model of barriers to and facilitators of engagement with remote measurement technology.

Many of the factors discovered are consistent with the engagement attributes previously reported by O’Brien and Toms [3] in their model for engagement with technology. They described a dynamic model, where engagement is a continual cycle of engagement, disengagement, and reengagement that persists over time. While they described many factors that drive engagement with technology in general, RMT to manage health outcomes is a specific and unique technology, in which health-related symptoms and potential moderators offered by health care providers should be considered. Building on this work and using themes from this review, we present a model of the most prominent influences on RMT engagement, including key facilitators (Figure 2).

Engagement in our model is moderated by health status, usability, convenience and accessibility, perceived utility, and motivation to engage. Engagement may be at its strongest when the user is able to use the technology, perceives the technology to be useful, and wants to use the technology.

#### Health Status

Of particular importance to RMT systems for management of health outcomes is the health status of the user. Health status will inevitably have an impact on what constitutes a usable, convenient, accessible, or valuable feature of an RMT system. As an example, being unwell and outside of one’s usual environment or routine (eg, in the hospital) led to disruptions in engagement and dropout [6,10,18]. However, some evidence suggests that people who were experiencing a higher level of problems (eg, greater physical or mental disability) engaged better [9,17]. While health severity and need for support may increase one’s motivation to participate, factors such as health condition and disability status, including typical or fluctuating symptoms, should always be considered in the design and implementation of RMT systems for management of health outcomes.

#### Usability

At the heart of this proposed model is usability. There may be individual differences that moderate usability, including variables such as age, past experience with technology, and exacerbations in health conditions and disability status, as well as the influence of how the system is designed. Problems with usability were the most common reasons for dropout from the studies. There is evidence that older adults were harder to engage [19,28,37]. This was partly because some were unfamiliar with using mHealth tools such as smartphone and wearable devices or did not feel motivated to learn new skills, but also because the devices were of unsuitable size to accommodate changing needs (eg, larger, more legible font sizes). Where content is presented clearly, such as in a smartphone app, and adequate support (actions or resources designed to help users work through challenges posed by the system) is offered, engagement seems to be facilitated [8].

However, the specific parameters for this support are unknown and need further research with clearly quantifiable outcomes. In addition, involvement of user experience methods is important for the development of usable mHealth tools for RMT systems in the future, with coproduction and user-centered design processes to validate choices [39].

#### Convenience and Accessibility

The need to be able to integrate the RMT system into a user’s normal routine was clear. Participants preferred tools that fit in with daily routines and tools that have already been adopted, with some disengaging and dropping out if unacceptable alternatives were offered. Personalization and demonstrating flexibility, in terms of taking into account the specific disabilities and needs of clinical groups, may be key in the design of usable RMT systems. This may include individual goal setting of dates and times for study activities, opting in or out of certain tracking activities (eg, reducing intrusiveness), or accommodating for health-related differing abilities. It may be important to note that forgetfulness emerged as a key barrier to engagement, which may suggest that the cognitive burden placed on individuals to remember to complete RMT schedules, in these studies, was too great. The value of notifications and reminders to carry out tasks has been demonstrated through usage statistics. That said, the magnitude of the effect varied between studies, with 1 study demonstrating a much bigger impact of notifications. This suggests that other factors moderate the likelihood of self-initiated engagement. Prompts have been mentioned to help aid memory, but there was some suggestion that the timing of these strategies may be important [17] and that there may be individual differences in preferences, with notifications that are too frequent being experienced as intrusive [32], thereby increasing cognitive burden. However, the studies did not manipulate these factors in an experimental design to test their impact, and this needs further research. Additional practical problems, such as poor Wi-Fi access, mobile data and network coverage, or compatible devices proved prohibitive to engagement [14,15,21]. Individual adaptation is exemplified by the size of devices. In some studies participants wanted smaller, more portable devices [31], and in other studies participants expressed the desire to have bigger monitors to be able to see their health data and complete the surveys more easily [19]. Balancing these goals may be a challenge for the development of future resources and may require coproduction with users to determine what is acceptable given a specific context. Some flexibility may be possible, for example, the use of responsive app designs that scale to the device being used. However, with the likelihood of large individual variation, this will be a major challenge for implementing RMT. Further research is needed to better quantify the magnitude of other potential facilitators that may help to overcome the barriers associated with convenience and accessibility.

#### Perceived Utility

We propose that increasing the rewards of using RMT increases the overall perceived value of the system in the face of some potential costs. Costs included financial costs of purchasing equipment, as well as concerns about privacy and reliability or accuracy of the data collected. As a strategy for increasing rewards associated with RMT systems, feedback is generally accepted, tolerated, and, in some cases, actively sought by users of RMT systems. In this context, feedback is considered to be additional information that participants receive from an RMT system about their health, their participation, or the larger program from which users and participants can derive value. This could include health information, rates of participation or adherence, metrics defined in goal-setting exercises, positive reinforcements, or general information about the study or their health condition. It was commonly reported that participants would like to receive more feedback [17,22,26], with some concluding that future efforts to improve long-term engagement should include positive reinforcements [10]. There is some emerging evidence for a role of social comparison and of incentives through gamified competition and monetary rewards on maintaining engagement. What is not yet known is what is the most effective method of providing feedback and incentives, and it may be important to note that perceptions of reward may differ between individuals. People with more severe health problems may be more likely to engage with RMT. This may be linked to perceived utility, as people with worse health status may perceive greater potential benefit to using the RMT.

#### Motivation

Motivation was a smaller but important category emerging from the analysis of the results of previous studies using RMT systems for the management of health outcomes. Without motivation, participants may not engage with the initial process of learning how to use a new system, and this category is inextricably linked to all other factors discussed previously. Even if users are familiar with mHealth tools such as smartphones and wearable devices, they may need additional motivation to integrate a new set of behaviors, such as responding to surveys. Lack of motivation is therefore a fundamental barrier to engagement. The factors presented thus far should be considered not just at the initiation of the study, but also as engagement is managed over time, because perceptions of the technology’s value or usability may change with prolonged use (eg, if expectations are not met). Therefore, we recommend steps to increase, or mitigate decreased, motivation with an RMT system to maintain motivation, and therefore engagement, over time.

### Limitations of Previous Research and Future Recommendations

Facilitators identified include convenience and accessibility, perceived utility, and motivation, but these factors are drawn from of pool of studies that varied greatly in terms of their quality. In addition, we conceptualized engagement as a process that should include disengagement and reengagement when required, but most findings reported in the studies included in this review relate to moderators of initial and sustained engagement. Although in our model we tentatively propose a feedback loop between the point of disengagement and the same barriers and facilitators affecting initial and sustained engagement, it is possible that factors affecting reengagement may be different, and this was not the focus of the studies. Future research should focus on the entire engagement process and quantify the impact of specific variables on engagement in terms of observable changes in usage statistics in rigorous experimental design. Some examples might be looking at the impact of different types of support (automated messages vs personalized messages vs direct human support) on the number of interactions and overall time spent using a smartphone app or wearable device. The impact of different types of feedback (immediate vs delayed vs no feedback) and data visualization or communication methods (graphs vs text messages vs discussion with a study coordinator) or environment (hospital vs home-based use) also need to be explicitly tested. Careful experimental manipulation is missing from the literature to date and, to be able to compare across these conditions, quantitative measures and usage statistics also require more standardization. A similar conclusion has also been drawn when considering adherence [40]. As a minimum, the number of interactions with apps (both total interactions and numbers of days) and time spent wearing devices relative to the length of the trial needs to be collected.

It is not enough for software developers to consider their systems in isolation from the individuals who may be using them. One of the main ways to develop engaged systems is to begin with codesign with those individuals who will be using the system. This is especially important for those involved in providing RMT for improving health. Before RMT systems are tested, there needs to be an iterative design process that explores acceptability, such as following the principles of user-centered design [41,42]. The feedback gathered may be qualitative, and some of this exploratory work has been conducted and forms the basis of the model we present in this paper. However, this work needs to lead into quantitative assessment as described above.

### Conclusions

The themes discovered in this review emerged across two different time periods providing validity information, but this evidence suggests that we are continuing to make the same mistakes. There is a great potential for RMT systems to augment and extend health care, but there remain clear challenges that still need to be overcome. Two suggestions are, first, to improve how we measure the impact of modifiable variables on engagement in order to understand the magnitude of effects. Second, several studies suggest working with the target users directly to coproduce systems that are acceptable and feasible to use over long periods of time. Our model indicates the interrelationship between key facilitators on the one hand, and the person and RMT factors on the other, that could act as a prototype for the development of RMT in the future.

## Appendix

Multimedia Appendix 1 Search strategy

Multimedia Appendix 2 Facilitators of and barriers to engagement in active RMT.

Multimedia Appendix 3 Facilitators of and barriers to engagement in passive and combination RMT.

Multimedia Appendix 4 Systematic review quotes

Multimedia Appendix 5 Characteristics of the original studies included in the systematic review.

## Abbreviations

MMAT: Mixed Methods Appraisal Tool
PRISMA: Preferred Reporting Items for Systematic Reviews and Meta-Analyses
RMT: remote measurement technology
