# Supplementary material for: Barriers to and Facilitators of Engagement With Remote Measurement Technology for Managing Health: Systematic Review and Content Analysis of Findings
Source: J Med Internet Res. 2018 Jul 12;20(7):e10480. doi: 10.2196/10480 (PMC6062692; doi:10.2196/10480)
Supplement: Multimedia Appendix 1 [file jmir_v20i7e10480_app1.pdf]

Appendix 1. Search strategy for barriers and facilitators to engagement with RMT.

Search line	Search terms	Filtered by
1	Exp Telemedicine/	Title/Abstract
2	((remot$ or distance$) adj5 (monitor$ or sens$ or track$ or check$))	Title/Abstract
3	(mobil$ adj3 (comput$ or techno$ or health$ or devic$))	Title/Abstract
4	(m-health or mhealth or ((digital$ or pervas$) adj2 health$))	Title/Abstract
5	((1 or 2 or 3 or 4) adj5 (facilitat$ or barrier$ or challenge$ or engag$ or accept$ or feasib$)	Title/Abstract
