# Supplementary material for: Barriers to and Facilitators of Engagement With Remote Measurement Technology for Managing Health: Systematic Review and Content Analysis of Findings
Source: J Med Internet Res. 2018 Jul 12;20(7):e10480. doi: 10.2196/10480 (PMC6062692; doi:10.2196/10480)
Supplement: Multimedia Appendix 2 [file jmir_v20i7e10480_app2.pdf]

Facilitators and barriers to engagement in Active RMT.

| | | Original articles 2014-2017<br>Reported by service users | | | | | | | | | | | | | | | | | |
|---|---|---|---|---|---|---|---|---|---|---|---|---|---|---|---|---|---|---|---|
| | **Factors** | | | | | **Dicianno et al (2016)** | **Engelhard et al (2017)** | | | | | | | | | | | | **Westergaard et al (2017)** |
| **Health status** | Exacerbations in condition, eg, requiring hospitalization | | | | | | | | | | - | | | | | | | | |
| | Difficulties with vision | | | | | | | | | | | | | | | | | - | |
| **Usability** | Clear, simple, and informative | | + | | | | | | | | | | | | | | | | |
| | Short battery life | | | | | | | | | | - | | | | | | | | |
| | Technical malfunctions | | | | | | | - | | | - | - | - | | | | | - | |
| | Speed of system | | | | | | | | | | | | | | | | | | |
| | Size of screen or device | | | | | | | | | + | | | | | | | | | |
| | Lost or damaged devices | | - | - | | | | | | | | | | | | | | | |
| | Changes to service plans | | | | | | | | | | | | | | | | | - | |
| **Convenience and accessibility** | Passive data collection | | | | | | | | | | | | | | | | | | |
| | Forgetfulness of the user | | | | | | | | | | - | | | | | | | | |
| | Presence and timing of notifications | | | | | +/- | | | | | | | | | + | + | | | |
| | Familiarity and knowledge | | | | + | | | | | | | | | | | | | | + |

| | | | | | | | | | | | | | | | | | | | |
|---|---|---|---|---|---|---|---|---|---|---|---|---|---|---|---|---|---|---|---|
| | Fits with routine | | | | | | | | | | | | | | | | | | |
| | Limited/inconvenient placement | - | | | | | | | | | | | | | | | | | |
| | Access to internet connection | | | | + | + | | | | | + | | | | | | | | |
| | Poor phone network coverage | | | | | | | | | | | | | | | | - | | |
| | Tailored or personalized | | | | | | + | | | | | | | | | | | | |
| | Available training or support | | | | | | + | | | + | | | | | | | | | |
| **Perceived utility & value: rewards** | Feedback | | | | + | | | | | | | | | | | | | | |
| | Incentives or social comparison | | | | | | | | + | | | | | | | | | | |
| **Perceived utility & value: costs** | Financial costs | | | | | | | | - | | | | | | | | | | |
| | Impact on privacy or security | | | - | | | | | | | | | | | | | | | |
| | Poor data reliability | | | | | | | | | - | | | | | | | | | |
| **Motivation** | Lack of intrinsic motivation to learn or use | | | | | | | | | | | | | | | | | | |
| | Influence of clinicians/extrinsic motivation | | | | | | | | | | | | | + | | | | | |
