# Supplementary material for: Barriers to and Facilitators of Engagement With Remote Measurement Technology for Managing Health: Systematic Review and Content Analysis of Findings
Source: J Med Internet Res. 2018 Jul 12;20(7):e10480. doi: 10.2196/10480 (PMC6062692; doi:10.2196/10480)
Supplement: Multimedia Appendix 3 [file jmir_v20i7e10480_app3.pdf]

Facilitators and barriers to engagement in passive RMT (gray) and combination RMT (white).

		Original articles 2014-2017<br>Reported by service users														
	**Factors**	**Anderson et al (2016)**	**Aranki et al (2016)**	**Buchem et al (2015)**	**Cushing et al (2016)**	**Ding et al (2016)**	**Eisenhauer et al (2017)**	**Evangelista et al (2015)**	**Fontil et al (2016)**	**Hardinge et al (2015)**	**Maglalang et al (2017)**	**Naslund et al (2015)**	**Peng et al (2016)**	**Randriambelonoro et al (2017)**	**Spring et al (2017)**	**Vathsangam et al (2014)**
**Health status**	Exacerbations in condition, eg, requiring hospitalization		-							-						
	Difficulties with vision							-								
**Usability**	Clear, simple and informative						+									
	Short battery life					-										
	Technical malfunctions				-				-	-	-			-	-	
	Speed of system									-						
	Size of screen or device															
	Lost or damaged devices						-							-		
	Changes to service plans															
**Convenience and accessibility**	Passive data collection	+														
	Forgetfulness of the user								-			-		-		
	Presence and timing of notifications				+											
	Familiarity and knowledge								+				+	+		
	Fits with routine					+							+			
	Limited/inconvenient placement															

	Access to internet connection						+		+							
	Poor phone network coverage						-									
	Tailored or personalized										+					
	Available training or support								+							
**Perceived utility & value: rewards**	Feedback	+		+												+
	Incentives or social comparison	+		+	+							+				
**Perceived utility & value: costs**	Financial costs											-				
	Impact on privacy or security															
	Poor data reliability						-									
**Motivation**	Lack of intrinsic motivation to learn or use	-						-					-			
	Influence of clinicians/extrinsic motivation												-			
