# Supplementary material for: Barriers to and Facilitators of Engagement With Remote Measurement Technology for Managing Health: Systematic Review and Content Analysis of Findings
Source: J Med Internet Res. 2018 Jul 12;20(7):e10480. doi: 10.2196/10480 (PMC6062692; doi:10.2196/10480)
Supplement: Multimedia Appendix 4 [file jmir_v20i7e10480_app4.pdf]

Systematic Review – Quotes

Feedback	
Anderson (2016)	“if it just means that I have to go move that little bit more, or I have to exercise that little bit more’, I will do it, because you have a real-time gauge of how well you've done for the day. So that gets me going because the perceived barrier of just getting the thing done is a lot lower.”
Buchem et al (2014)	“50% of participants expressed a higher understanding of an effective training due to badges and stated that badges helped them to get a better overview of their training progress. The survey also results show that 50% of participants felt motivated by badges during the study period”
Dale et al (2015	“Participants revealed that the text messages were motivating and acted as an incentive and a reminder to exercise… 67% liked motivational text messages”
Vathsangam & Sukhatme (2014)	“The second most important feature was talking about the application data with the study coordinator. This suggests that it is not simply the data that is important but the application plus some contextual insight about lifestyles that matters to this demographic and the ability to interpret the data is important. This has implication on how these data are presented so as to provide contextual insights.”
Incentives or social comparison	
Anderson et al (2016)	“With one exception [p8], all reported increased engagement when describing the social or competitive angle of their app by all participants”
Buchum et al (2015)	"Overall, 80% of participants stated that they had a lot of fun when training and engaging with the fMOOC. Especially the training instructions and the wearable tracker were seen as important factors for motivation."
Cushing et al (2016)	“Interviewed patients liked the gaming aspects of the mobile app and felt that the point system and monetary rewards motivated them to use their medication. Two patients suggested adding more features such as unlocking in-app customization of the hero avatar and “mini games” with continued use.”
Ho et al (2015)	“Participants felt that the program provided the right amount of accountability, incentive and support to allow them to achieve their goals”<br>“Sharing insights on the social media platform provided opportunities to learn from others, share and celebrate success, and created a sense of accountability and community”
Naslund et al (2015)	“Several participants stated that they enjoyed seeing each other’s steps on the smartphone application as a form of competition and motivation to do more activity”
Financial Costs	
Ho et al (2015)	“more than half of the participants (56%) also indicted that based on their current income they would be either unable to afford the program, or that they would find it to be a significant financial burden”
Naslund et al (2015)	“Participants viewed these devices as expensive and difficult to obtain for low-income individuals”
Impact on privacy/security	
Ben-Zeev et al (2016)	“One participant reported getting paranoid about the mobile phone and breaking it. Another participant reported only using it on “airplane mode” to avoid being tracked. Three participants deleted the FOCUS program from the phone.”
Poor data reliability	
Eisenhauer et al (2017)	“Time and difficulty with locating exact food-portion sizes were reasons the men cited on the survey for discontinuing logging…“I went kayaking and didn’t get any credit for movement in the kayak”
Huang et al. (2015)	"50% of the respondents were uncertain about the user benefits, were not convinced that it was reliable for data entry, and did not wish to continue to use such a device."

Lack of motivation to learn/use	
Anderson et al. (2016)	“Inability to engage with one’s health app can result in declined usage: “I do have some apps I don't use often, mainly because they've kind of bored me in a way.”
Evangelista et al. (2015)	“Three of the older participants indicated that their family members helped them with daily transmission because they could not see the visual display or did not want to learn how to use the RMS”
Peng et al (2016)	“The last barrier was the required time and effort to use the apps for self-monitoring. One participant stated, ‘‘The only thing on the negative side is that [the apps] take a lot of input, all the time [it takes] to put all the food in’’ (female 62). One female participant who had a diabetes self-management app shared that using the app over time became burdensome and annoying because it repeatedly asked her to enter information.”
Influence of reception from clinicians	
Lind et al (2016)	“After the completion of the study and because of demand from the professional caregivers, the system was in continued use during 18 months, after which a new intervention study started”
Peng et al (2016)	“Fourth, some participants did not use any tools for self- management because their doctor did not ask them to. Many of them expressed that they wanted recommendations from their healthcare providers regarding health apps.”
Passive data collection	
Anderson et al (2016)	“I use . . . [certain health apps] because they're connected to wearables, so I don't have to do a<br>lot of the data collection. It does it automatically for me and then feeds me back the information.”
Presence and timing of notifications/messages	
Cushing et al (2016)	“All three mobile app users who completed the study agreed that the app helped remind them to take their medication, that they would be interested in continuing to use the mobile app beyond the study period”
Dicianno et al (2016)	"Participants met or exceeded set benchmarks for use and were more likely to use modules that reminded them to conduct activities that occurred on a less than daily basis…Some participants had already incorporated these activities into their daily routine and felt they did not need such frequent reminders"
McClure et al (2016)	“Of the 168 check-in surveys completed, 11 were self-initiated and the remainder were in response to a reminder prompt.”
Cushing et al (2016)	“They reported receiving multiple reminder messages during the day when they were at school and did not have access to their phones. They agreed they would like less frequent and shorter messages.”
Mundi et al (2015)	“modifications could be made to make the messaging more ‘flexible’. For instance, a ‘snooze’ feature was requested by several subjects, which would have allowed them to answer a particular message as soon as they were able. Such a feature may enhance the flexibility of such technology, potentially increasing the EMA response”
Familiarity with devices	
Dale et al (2015)	“The second obstacle for some participants was lack of knowledge about how to use the Web site, in particular the log-on system.”
Fontil et al (2016)	“Even among those who completed the sign-up process with one-on-one assistance, we observed several computer literacy challenges for a majority of participants”
Peng et al (2016)	“Second, they faced a technical literacy barrier. One participant said, ‘‘I’ve never used it [these apps] because I never got it to work the way I wanted it to’’
Randriambelonoro et al (2017)	“We did not know how to use the dashboard on the computer. What we saw was not the same as that on the Fitbit screen. I was frustrated with the computer, and he was

	frustrated with the Fitbit. We are old and it is difficult"
Westergaard et al (2017)	"One sixty-two year-old woman reported the system was somewhat difficult in the beginning beacuse she had never used a smartphone before; by the end of the study she reported it was easy to use."
Fits with routine	
Ding et al (2016)	"We terminated the study for 2 participants (1 in the experimental group and 1 in the control group) because the WalkMore app was not compatible with their phones (Samsung S4)."
Peng et al (2016)	"Third, they did not use mobile apps because they found the tools they were using, such as a paper logbook or a glucometer, already satisfied their needs."
Limited/inconvenient placement	
Al Ayubi et al (2014)	"We identified that PersonA has some utility limitations, such as…limited placement of the devices"
Access to internet connection	
Dale et al (2015)	"The lack of high-speed broadband Internet to successfully use the program was a noted barrier, as described in the following quote… …my internet connection at home isn't strong enough to play the videos. And I can't do it at work really so that was why I didn't use the website."
Dicianno et al (2016)	"2 participants voluntarily withdrew (one in the intervention group could not acquire consistent wireless service"
Eisenhauer et al (2017)	"Barriers that delayed connectivity with the mHealth technology included old computer systems, low Internet connection bandwidth, and lack of Wi-Fi in the community."
Fontil et al (2016)	"Among those who did not complete the sign-up process, it was mostly due to technical issues with their computer or internet not working"
Jonassaint et al (2016)	"Five patients experienced some difficulty including poor Internet connection at home (n=3)"
Poor phone network coverage	
Eisenhauer et al (2017)	"Four men reported up to an 8-hr delay in receiving text messages due to weak cell phone signals"
Piotrowicz et al (2014)	"About 39% of patients missed doing an ET session (averagely one for whole HTCR cycle) because of the fact that the particular mobile phone network operator was unavailable."
Individually tailored or personalised	
Engelhard et al (2017)	"Free-response feedback points to an important contributing factor: the intervention was not adequately tailored to the disability status of individual sub- jects. Some subjects felt that questions were not relevant to them, while others grew tired of reporting the same unchanged symptoms."
Maglalang et al (2017)	"First, a culturally tailored intervention and bi-cultural Filipino research staff enhanced engagement…. Over half (n=26, 57.8%) of the respondents stated that the culturally tailored support in terms of materials and staff enhanced their engagement…The Fitbit database was limited and did not include Filipino foods. Participants reported this limitation made logging daily food/caloric intake a tedious task."
Technical support or facilitated learning	
Engelhard et al (2017)	"Only 12.9% of subjects had technical difficulty with the log-in process. In each case, this was solved by phone call with the study coordinator"
Fontil et al (2016)	"Just real guidance (would have helped me sign up)… I just got really frustrated"
Huang et al. (2015)	"These operating mistakes were a temporary phenomenon, which were easily corrected by offering the participants a few minor instruction, resulting in

	improvement in the operation of these devices"
Clear, simple, informative	
Ben-Zeev et al (2014)	“Over 90% of participants thought the different components of the intervention worked well together, that content appeared on the screen clearly, and that people could learn to use FOCUS very quickly.”
Eisenhauer et al (2017)	“Time (peak planting season) and difficulty with locating exact food-portion sizes were reasons the men cited on the survey for discontinuing logging.”
Short battery life	
Ding et al (2016)	“1 participant opted out of our study because he thought the installed apps consumed more battery and he was not able to charge the phone in time.”
Jonassaint et al (2016)	“Barriers to completing entries noted by patients included… short battery life of the device.”
Technical malfunctions	
Cushing et al (2016)	“Seven sensor devices were used in the study, and six successfully uploaded data to the HIPAA-compliant server. One user’s data were not uploaded because the patient was unable to sync the inhaler sensor to his iPad.”
Fontil et al (2016)	“Among those who did not complete the sign-up process, it was mostly due to technical issues with their computer or Internet not working.”
Hardinge et al (2015)	"there were technical problems affecting one patient’s use of the tablet on day 13 which, although resolved, led to the patient deciding to withdraw"
Hartzler et al (2016)	“Participants encountered minor problems creating user accounts, answering intake questions, and navigating content due to unexpected behavior of keyboards, scroll bars, buttons, and other interface widgets that could be addressed with minor adjustments implementing stands iOS interactions.”
Jonassaint et al (2016)	“Five patients experienced some difficulty including…and/or technical difficulties with the app that were: frequent crashing (n=1), repeated notification and over-alerting (n=1), and deletion of the app when a new iOS update was installed (n=1). Technical difficulties adversely impacted compliance such that the patients who experienced technical difficulties completed fewer daily entries (41.0%) than those who did not (76.0%, p=0.017).”
Juengst et al (2015)	“Overall, most technical problems were related to not receiving notifications, receiving notifications at the wrong time, the application freezing or the application not submitting the assessment.”
Leonard et al (2017)	“Data were unavailable for 2 children due to not having medication (n=1), or attrition (n=1), and limited to 1 to 2 video recordings for 2 children due to technology problems (n=2).”
Maglalang et al (2017)	“Besides the difficulties with the Fitbit app/diary, there were technological challenges. Some participants had problems using their smartphone or tablets, and synching their Fitbit to their online accounts.”
Price et al (2014)	"technical difficulties reported as the primary reason for non-response"
Randriambelonoro et al (2017)	“Some participants also encountered various technical problems but managed to use the essential functionality”
Spring et al (2017)	“However, the device sometimes lost power without warning and was difficult to restart, as is reflected by the greater coaching time utilized by participants in TECH as compared to STND.”
Speed of system	
Hardinge et al (2015)	“four others did not complete the study:… one patient disliked the system describing it as ‘too slow’.”
Size of screen or device	
Huang et al (2015)	“For the 10 participants able to use the 3.7-inchmHealth, a different response was

	seen in the category of ‘‘perceived ease of use.’’ Only 30% considered the device easy to use, and only10%agreed that it was reliable for data entry”
Jonassaint et al (2016)	“Patients using an iPad completed more daily entries (85.0%) than those with a smaller device, an iPhone or iPod Touch (55.0%, p<.0025).”
Lost or damaged devices	
Ben-Zeev et al (2014)	"One participant dropped out of the study after losing 2 study smartphones in the first week"
Ben-Zeev et al (2016)	“Another 21 participants reported their mobile phone lost or stolen over the course of their participation and requested a replacement device. One participant accidentally downloaded malware that rendered the phone inoperative and it needed to be replaced. At least two devices were pawned by participants.”
Eisenhauer et al (2017)	“Observed usage of the activity monitors use indicated that nine of the 12 men wore the monitor all 21 days, two wore it 9 and 15 days, respectively, and one lost the monitor…. Three men reported the device fell off during heavy labor involving stooping or squatting, such as fixing barbed wire fence, grinding hay, or carrying windows at waist level. Two men permanently lost their activity monitors by 6-weeks during these types of activities.”
Randriambelonoro et al (2017)	“although some users reported losing the device or forgetting to wear it.”
Changes to service plans	
Price et al (2014)	“Technical difficulties included: changing their service plan such that they were unable to receive text messages"
Exacerbations in condition, e.g. requiring hospitalisation	
Aranki et al. (2014)	"Note that in some cases, patients were rehospitalized during the course of the study and their usage dropped to zero."
Hardinge et al. (2015)	“four others did not complete the study:… two patients had multiple hospital admissions and became too unwell”
Jonassaint et al. (2015)	“Barriers to completing entries noted by patients included: being in a pain crisis”
Forgetfulness of the user	
Fontil et al (2016)	“However, while logins overall were high, a few participants expressed technology accessibility barriers that mirrored their challenges in completing the sign-up process, such as not being able to remember their passwords to get back into the online content”
Jonassaint et al. (2015)	“Barriers to completing entries noted by patients included:… being too busy and forgetting”
Naslund et al. (2015)	“Some participants voiced frustration if they forgot to sync their device with the smartphone or if they forgot to wear it.”
Randriambelonoro et al (2017)	“Of the two users who stopped wearing the device, one became so busy that he forgot to wear the tracker after the first two weeks.”
Difficulties with vision	
Evangelista et al. (2015)	"71% expressed the desire to have a bigger monitor to see their health data and complete the surveys…Three of the older participants indicated that their family members helped them with daily transmission because they could not see the visual display"
