# Supplementary material for: Barriers to and Facilitators of Engagement With Remote Measurement Technology for Managing Health: Systematic Review and Content Analysis of Findings
Source: J Med Internet Res. 2018 Jul 12;20(7):e10480. doi: 10.2196/10480 (PMC6062692; doi:10.2196/10480)
Supplement: Multimedia Appendix 5 [file jmir_v20i7e10480_app5.pdf]

Characteristics of the original studies included in the systematic review.

Sources of reported barriers/facilitators:

■ Quantitative Sources ■ Qualitative Sources □ Mixed Method Sources

First author, year, reference	Country	Population	Participant Characteristics	Description of RMT device	Type of RMT	Length of trial	Quantitative Sources	Qualitative Sources	Quality assessment (0 - 1)
Aranki, 2016 [18]	USA	Chronic Heart Condition	Male (n = 8)<br>Female (*n* = 7)<br>Age not specified	A smartphone app encompassing internal sensors to monitor energy expenditure, and daily surveys of cardiovascular symptoms (e.g. fatigue and dizziness) and vital signs (e.g. heartbeat rate and blood pressure)	Active and Passive	6 weeks	Usage statistics; Questionnaire	N/A	0.25
Ben-Zeev, 2014 [8]	USA	Psychosis	Male (*n* = 20)<br>Female (*n* = 13)<br>Age (Mean = 45.9, SD = 8.78)	*FOCUS*: smartphone apps and online dashboard using ecological momentary assessment (EMA) of current status (e.g. mood) with tailored feedback and interventions	Active	1 month	Usage statistics; Questionnaire	N/A	0.75
Ben-Zeev, 2016 [13]	USA	Psychosis	Male (*n* = 213)<br>Female (*n* = 129)<br>Age: 18-60	*FOCUS*: smartphone apps and online dashboard using ecological momentary assessment (EMA) of current status (e.g. mood) with tailored feedback and interventions	Active	6 months	Usage statistics	N/A	0.75
Buchem, 2015 [34]	Germany	Maintaining health and well-being amongst older adults	Male (*n* = 10)<br>Female (*n* = 10)<br>Age not specified	*fMOOC*: mobile app and pedometer, with educational and gamification (e.g. badges) components	Passive	4 weeks	Usage statistics; Questionnaire	N/A	0.25
Dicianno, 2016 [14]	USA	Spina Bifida	**Intervention**<br>Male (*n* = 8)<br>Female (*n* = 5)<br>Age (Mean = 29.7, SD = 5)<br><br>**Control**<br>Male (*n* = 5)<br>Female (*n* = 5)<br>Age (Mean = 29.5, SD = 6.8)	*iMHere:* mobile- and web-based PROMs (e.g. mood, physical independence and self-management skills), monitoring of medication adherence, and skin damage reports/image uploads	Active	12 months	Usage statistics	N/A	0.75

Ding, 2016 [29]	USA	Maintaining health and well-being	Male (*n* = 10)<br>Female (*n* = 6)<br>Age: 18-25	*WalkMore:* mobile- and smart watch-based monitoring of physical activity, with motivational reminders	Passive	4 weeks	Questionnaire	N/A	0.5
Engelhard, 2017 [9]	USA	Multiple Sclerosis	Male (*n* = 2)<br>Female (*n* = 29)<br>Age: (Median = 48, 27-61)	Web-based PROMs (e.g. fatigue, walking impairment and leisure-time exercise habits) and web portal	Active	6 months	Usage statistics	N/A	0.75
Hardinge, 2015 [6]	UK	COPD	Male (*n* = 9)<br>Female (*n* = 9)<br>Age: 40-90	Tablet-based daily symptom diaries assessing general well-being, cough and sputum production (quantity and color), and breathlessness, a wireless pulse oximeter, and personalized self-management plans	Active and passive	6 months	Usage statistics	N/A	1
Huang, 2015 [28]	Taiwan	Maintaining health and well-being amongst older adults	Male (*n* = 29)<br>Female (*n* = 22)<br>Age: 50-94	Mobile-pad and smartphone-based assessments of physical (e.g. hearing, speech, vision and nutrition) and mental (e.g. cognition and mood) health status	Active	6 weeks	Questionnaire	N/A	0.25
Jonassaint, 2015 [10]	USA	Sickle Cell Disease	Male (*n* = 9)<br>Female (*n* = 6)<br>Age: (Mean = 29, 16-54)	*SMART:* smartphone-based recording of pain intensity and clinical symptoms	Active	28 days	Usage statistics	N/A	0.5
Juengst, 2015 [23]	USA	Traumatic Brain Injury	Male (*n* = 12)<br>Female (*n* = 8)<br>Age: (Mean = 36.7, SD = 12.4, 22-60)	*iPerform:* smartphone-based EMA of affect, anxiety and fatigue	Active	8 weeks	Questionnaire	N/A	0.75
Leonard, 2017 [24]	USA	Sickle Cell Disease and β-thalassemia major	**Patients**<br>Male (*n* = 4)<br>Female (*n* = 7)<br>Age: (Mean = 12.4, SD = 3.8, 8-21)<br>**Caregivers**<br>*n* = 1 (gender and age not specified)	Self-recorded videos of therapy (iron chelation) administration, uploaded to a remote service via a smartphone or tablet app	Active	6 months	Usage statistics	N/A	0.75
Lind, 2016 [38]	S3weden	Heart Failure	Male (*n* = 11)<br>Female (*n* = 3)<br>Age: (Mean = 84, SD = 5.6, 75-95)	Daily PROMs (e.g. shortness of breath, weight, blood pressure and oxygen saturation) recorded via digital pen-and-paper	Active	13 months	Questionnaire	N/A	0.5

McClure, 2016 [12]	USA	Smoking Cessation	**Intervention**<br>Male (*n* = 14)<br>Female (*n* = 19)<br>Age: (Mean = 48.4, SD = 8.4)<br>**Controls**<br>Male (*n* = 15)<br>Female (*n* = 18)<br>Age: (Mean = 50.6, SD = 8.9)	*MyMAP*: check-in surveys to report current symptoms and side effects completed via a mobile-based program	Active	5 months	Usage statistics; Questionnaire	N/A	0.5
Mundi, 2015 [33]	USA	Pre-bariatric Surgery	**At enrolment**<br>Male (*n* = 3)<br>Female (*n* = 27)<br>Age: (Mean = 41.3, SD = 11.4)<br><br>**Study Completion**<br>N = 20 (gender and age not specified)	Smartphone-based EMA of physical activity and dietary intake, with motivational and educational components	Active	12 weeks	Questionnaire	N/A	0.5
Piotrowicz, 2014 [7]	Poland	Cardiovascular Disease	Male (*n* = 307)<br>Female (*n* = 58)<br>Age: (Mean = 58.3, SD = 10.5)	Tele-electrocardiogram (ECG)-monitoring transmitted via a mobile phone to a monitoring center	Active	4 weeks	Usage statistics; Questionnaire	N/A	1
Price, 2014 [17]	USA	PTSD	Male (*n* = 17)<br>Female (*n* = 14)<br>Age: (Mean = 37.1, SD = 9.8)	Daily SMS-based assessments of social support, hypervigilance, avoidance, re-experiencing and pain	Active	15 days	Usage statistics; Questionnaire	N/A	0.5
Spring, 2017 [27]	USA	Obesity	Male (*n* = 15)<br>Female (*n* = 81)<br>Age: (Mean = 39.3, SD = 11.7)	Monitoring of dietary intake, body weight and physical activity via a smartphone app and accelerometer *(n=32)*	Active and Passive	6 months	Usage statistics	N/A	0.75
Anderson, 2016 [32]	Australia	Chronic health conditions and Maintaining health and well-being	Male (*n* = 7)<br>Female (*n* = 15)<br>Age: 18- >55	A variety of health apps for physical (e.g. blood pressure, diabetes and migraine) and mental (e.g. anxiety) health, with most requiring weekly interaction	Active and Passive	Single Session	N/A	Semi-structured interviews	0.75
Fontil, 2016 [21]	USA	Diabetes	Male (*n* = 8)<br>Female (*n* = 10)<br>Age: (Mean = 53)	*The Omada Health Program:* wireless weight scales and pedometer, with online group support and personalized coaching	Active and Passive	4 weeks	N/A	Focus Groups	0.75

Maglalan, 2017 [25]	USA	Type 2 Diabetes	Male (*n* = 17)<br>Female (*n* = 28)<br>Age: (Mean = 57.6, SD = 9.8)	*PilAm Go4Health:* physical activity, calorie intake and weight recorded via a Fitbit	Active and Passive	6 months	N/A	Semi-structured interviews	0.75
Peng, 2016 [30]	USA	Type 2 Diabetes	Male (*n* = 5)<br>Female (*n* = 13)<br>Age: (Mean = 54, SD = 12.7)	Four apps (Glucose Buddy, mySugr, MyFitnessPal and MapMyWalk) with features including activity tracking, goal setting and peer support	Active and Passive	Single Session	N/A	Focus Groups	0.75
Randriambelonoro, 2017 [26]	Switzerland	Diabetes and Obesity	Male (*n* = 7)<br>Female (*n* = 11)<br>Age: 36-73	*FitBit One:* activity monitor, daily food log and SMS reminders	Active and Passive	7 months	N/A	Semi-structured interviews	0.5
Westergaard, 2017 [37]	USA	HIV and Substance Use Disorders	**At enrolment**<br>Male (*n* = 12)<br>Female (*n* = 7)<br>Age: (Median = 49.3, IQR = 45-54.6)<br>**Study Completion**<br>N = 15 (gender and age not specified)	*mPeer2Peer:* smartphone-based EMA of symptoms and behaviors (e.g. drug and alcohol cravings, usage and mood)	Active	9 months	N/A	Semi-structured interviews	0.75
Al Ayubi, 2014 [31]	USA	Maintaining health and well-being	Male (*n* = 3)<br>Female (*n* = 10)<br>Age: (Mean = 32.2, SD = 5.6, 24-45)	*PersonA*: smartphone with internal accelerometer to monitor physical activity, and online platform (Facebook) for social support	Passive	4 weeks (social interaction introduced in week two)	Questionnaires	Semi-structured interviews	0.25
Cushing, 2016 [20]	USA	Asthma	Male (*n* = 2)<br>Female (*n* = 5)<br>Age: (Mean = 14.1, 11-18)	*Mobile asthma management system:* an inhaler sensor and mobile app to monitor medication adherence and send reminders	Passive	12 weeks	Usage statistics	Focus Groups	0.5
Dale, 2015 [11]	New Zealand	Chronic Heart Condition	Male (*n* = 69)<br>Female (*n* = 16)<br>Age: (Mean = 61.4, SD = 9)	Step count monitoring via a pedometer, with goal setting and motivational components via text messaging and a web site	Active	24 weeks	Usage statistics; Questionnaire	Semi-structured Interviews	0.75
Eisenhauer, 2017 [15]	USA	Maintaining health and well-being in rural men	Male (*n* = 12)<br>Age: 40-66	*FitBit One:* wearable activity monitor and companion mobile app to record daily dietary intake, and issue SMS reminders	Active and Passive	3 weeks	Usage statistics; Questionnaire	Semi-structured Interviews	0.5
Evangelista, 2015 [19]	USA	Chronic heart problems	Male (*n* = 10)<br>Female (*n* = 11)<br>Age: (Mean = 73.1, SD = 9.2)	Monitoring of physical health parameters (e.g., weight, blood pressure, and heart rate) via a wireless device, daily symptom assessment questionnaires via SMS, disease related alerts and	Active and Passive	12 weeks	Usage statistics	Semi-structured Interviews	0.5

				reminders, and computer assisted personal interviews					
Hartzler, 2016 [22]	USA	Type-2 Diabetes or Major Depressive Disorder	**Diabetes**<br>Male (*n* = 2)<br>Female (*n* = 6)<br>Age: (Mean = 71, SD = 5, 63-77)<br>**Depression**<br>Male (*n* = 5)<br>Female (*n* = 2)<br>Age: (Mean = 63, SD = 12, 36-73)	*NutriWalking:* mobile-based activity and nutrition log, with personalized goals and peer support	Active	Single Session	Questionnaire	Semi-structured interviews	0.5
Ho, 2015 [36]	Canada	Type-2 Diabetes	**Patients (*n* = 39)**<br>Gender and age not specified)<br>**Caregivers (*n* = 28)**<br>Gender and age not specified)	mDAWN: wireless blood pressure monitor, weight scale and glucose meter, with educational and social support via SMS and a web-portal	Active	3 months	Questionnaire	Semi-structured interviews	0.25
Naslund, 2015 [16]	USA	Serious Mental Illness	Male (*n* = 1)<br>Female (*n* = 9)<br>Age: (Mean = 47.7, SD = 9, 30-58)	Wearable activity tracking/accelerometer devices (FitBit Zip or Nike Inc. FuelBand) wirelessly synced to smartphone devices	Passive	Between 80 and 133 days	Usage Statistics	Semi-structured interviews	0.25
Vathsangam, 2014 [35]	USA	Maintaining health and well-being amongst older adults	Female (*n* = 8)<br>Age: (Mean = 74, SD = 11, 61-88)	*Strive:* physical activity monitored via an accelerometer smartphone app	Passive	3 weeks	Usage Statistics; Questionnaire	Focus Group	0.25
